# Protective effect of 3-hydroxybutyrate against endoplasmic reticulum stress-associated vascular endothelial cell damage induced by low glucose exposure

**DOI:** 10.1371/journal.pone.0191147

**Published:** 2018-03-19

**Authors:** Eri Soejima, Tsuyoshi Ohki, Yayoi Kurita, Xiaohong Yuan, Kayo Tanaka, Satomi Kakino, Kento Hara, Hitomi Nakayama, Yuji Tajiri, Kentaro Yamada

**Affiliations:** Division of Endocrinology and Metabolism, Department of Medicine, Kurume University School of Medicine, Kurume, Fukuoka, Japan; Duke University School of Medicine, UNITED STATES

## Abstract

**Aims/Hypothesis:**

The aim of this study was to elucidate the mechanism by which severe hypoglycemia accelerates vascular complications. Furthermore, we assessed the possible protective effect of ketone bodies against the endothelial cell damage caused by glucose deficiency.

**Methods:**

Human umbilical vein endothelial cells (HUVECs) were cultured at a glucose level of either 0.56 or 5.6 mmol/L with or without 3-hydroxybutyrate (3-HB) supplementation. Cell viability was assessed with a CCK-8 assay and a lactate dehydrogenase (LDH) release assay. The activity of caspases was measured using fluorogenic substrates. The expression of genes associated with endothelial cell function and endoplasmic reticulum (ER) stress was evaluated by real-time quantitative PCR. Protein levels of ER stress-related molecules were assessed by Western blotting.

**Results:**

Culture of HUVECs in low-glucose medium for 24 or 48 h resulted in reduction of cell viability accompanied by activation of caspase-3/7 and caspase-8. The addition of a pan caspase inhibitor attenuated the cell death. After incubation in the low-glucose medium, we found reduced mRNA and protein levels of endothelial nitric oxide synthase. ER stress responses mediated by phosphorylation of protein kinase RNA-like ER kinase (PERK) and cleavage of activating transcription factor 6 (ATF6) were augmented, but X-box binding protein 1 (*Xbp1*) splicing was reduced. Most of these responses to glucose deficiency were significantly attenuated by supplementation with 3-HB.

**Conclusions/Interpretation:**

These observations showed that exposure to low glucose induces ER stress, caspase activation, endothelial cell dysfunction and cell death. The beneficial effects of 3-HB shown in this study suggest that hypoketonemic severe hypoglycemia induced by insulin injections or insulin secretagogue administration may be more harmful than hyperketonemic severe hypoglycemia.

## Introduction

Severe hypoglycemia has been identified as a strong predictor of cardiovascular events in patients with type 2 diabetes. A meta-analysis of six eligible studies with 903,510 participants showed a relative risk of 2.05 for cardiovascular disease associated with severe hypoglycemia [1]. Severe hypoglycemia stimulates sympathetic neural activity, accompanied by a surge in plasma catecholamines, leading to vasoconstriction and platelet aggregation [2, 3]. However, recurrent hypoglycemia attenuates sympathoadrenal responses to hypoglycemia by a phenomenon called hypoglycemia-associated autonomic failure [4]. Thus, hypoglycemic attacks likely accelerate the development of vascular complications without the catecholamine surge in patients with diabetes.

It has been established that endothelial dysfunction characterized by the impairment of endothelium-dependent vasodilation is an early step in the development of atherosclerosis [5, 6]. The impairment is attributable to the reduction of nitric oxide (NO) bioavailability resulting from augmented oxidative stress. Furthermore, endothelial cell injury and apoptosis facilitate local lipid deposition and cause plaque instability [7]. Thus hypoglycemia-induced vascular damage could be associated with the impairment of NO production.

Glucose deficiency has been shown to increase endoplasmic reticulum (ER) stress in neurons and various cancer cells, resulting in the augmentation of autophagy and apoptosis [8,9]. For vascular endothelial cells, a number of studies have shown that oxygen-glucose deprivation, as a model of ischemic stroke, induced autophagy and apoptosis through oxidative stress [10–12]. However, the effects of glucose deficiency alone on endothelial cells have not been well described, and the role of ER stress in low glucose-induced endothelial cell damage remains to be determined.

When glucose is deficient, fatty acids and ketone bodies are used as energy sources. Unlike fatty acids, ketone bodies cross the blood-brain barrier and therefore can provide an alternate fuel source for the brain during starvation [13]. The utilization of ketone bodies requires β-ketoacyl-CoA transferase, which converts acetoacetate to acetoacetyl-CoA. This enzyme is present in all tissues except the liver, in which ketone bodies are generated. Therefore, ketone bodies likely also protect non-neural tissues against glucose deprivation. For example, ketone bodies are efficiently used in cardiac tissue as a fuel source when glucose metabolism is disturbed [14]. In fact, increased ketogenesis has been implicated in the favourable effects of a sodium glucose cotransporter 2 (SGLT2) inhibitor shown in the EMPA-REG OUTCOME study [15]. In the present study, we assessed the effect of ketone body supplementation on endothelial cell injury induced by glucose deficiency.

## Materials and methods

### Culture of human umbilical vein endothelial cells

Human umbilical vein endothelial cells (HUVECs) were purchased from Lonza (Walkersville, MD, USA) and maintained at 37°C under a humidified atmosphere of 95% air and 5% CO_2_ in endothelial growth medium (Lonza). For all experiments, HUVECs were used within passage seven. The cells were seeded at a density of 5 × 10^4^ cells/well in 96-well plates or 10 × 10^4^ cells/well in 24-well plates. Then, the cells were incubated in Medium199 (United States Biological, Salem, MA, USA) supplemented with 0.2% fetal bovine serum (Lonza) and 0.1% gentamicin sulfate/amphotericin-B (GA-1000, Lonza) at a glucose level of either 5.6 or 0.56 mmol/L with or without D-3-hydroxybutyrate (3-HB) (Sigma-Aldrich, St. Louis, MO, USA).

### Cell viability measurement

Cell viability was measured with a CCK-8 assay (Dojindo, Kumamoto, Japan) according to the manufacturer’s protocol. Briefly, 10 μl of CCK-8 solution was added to each well of a 96-well plate and incubated at 37°C for 4 h. The optical density was measured at an absorption wavelength of 450 nm. Results were normalized to control levels. Cell death was also quantified by a lactic acid dehydrogenase (LDH) release assay. LDH activity was assessed by determining the amount of NADH generated in a reaction between NAD(+) and lactate (L-Type LD. J, Wako, Osaka, Japan).

### Caspase-8 activity assay

The activity of caspase-8 was measured using the fluorogenic substrate Ac-LETD-AFC (Enzo Life Sciences, Farmingdale, NY, USA). Briefly, cells were lysed in lysis buffer containing 20 mmol/L Tris at pH 7.5, 150 mmol/L NaCl, 1 mmol/L EDTA, and 1% Triton X-100. Cell lysates were incubated with the fluorogenic substrate at a final concentration of 100 μmol/L in the dark at 37°C for 2 h. Caspase-8 activity was assessed by fluorescence emission at 517 nm with excitation at 400 nm using a microplate reader (EZS-FL, Asahi Techno Glass, Tokyo, Japan). The protein concentration in cell lysates was determined using a BCA Protein Assay Kit (Thermo Fisher Scientific, Waltham, MA, USA). Bovine serum albumin was used as a standard.

### Caspase-3/7 activity assay and assessment of the effect of a caspase inhibitor

Detection of caspase-3/7 was performed using a fluorogenic, no-wash indicator of activated caspase-3/7 (CellEvent Caspase-3/7 Green Readyprobes Reagent, Thermo Fisher). HUVECs were seeded on a 35 mm glass bottom dish. Then, the cells were incubated at a glucose level of either 5.6 or 0.56 mmol/L with or without 3-HB. After 24 h, cells were loaded with the reagent. Nuclei were visualized by Hoechst 33342 (NucBlue Live Cell Stain, Thermo Fisher, Waltham, MA, USA). Positive cells were obtained with a fluorescence microscope (Eclipse Ti, Nikon, Tokyo, Japan) and analysed with CellInsight CX5 (Thermo Fisher).

A pan caspase inhibitor zVAD-fmk (Selleck, Osaka, Japan) was added at the concentration of 10 μmol/L at the start of the low-glucose culture. After 24 h, cell viability was assessed with the CCK-8 assay and the LDH release assay.

### Real-time quantitative RT-PCR

The gene expression of endothelial nitric oxide synthase (eNOS), protein kinase RNA-like ER kinase (PERK), activating transcription factor 6 (ATF6), inositol-requiring enzyme 1 (IRE1), CCAAT-enhancer-binding protein homologous protein (CHOP), 78 kDa glucose-regulated protein (GRP-78), protein phosphatase 1 regulatory subunit 15A (PPP1R15A), and the unspliced and spliced forms of X-box binding protein 1 (XBP1) mRNA were measured by real-time quantitative PCR. Total RNA was extracted from HUVECs using a kit (RNAeasy micro, QIAGEN, Tokyo, Japan). cDNA was synthesized from 500 ng of total RNA with a PrimeScript RT reagent kit and gDNA Eraser (Takara, Tokyo, Japan). Gene expression was determined by quantitative RT-PCR using the SYBR green-based fluorescence method (SYBR Premix Ex Taq, Takara). Amplification was performed using StepOnePlus (Applied Biosystems, Foster City, CA, USA). The PCR cycling conditions were 30 sec at 95°C followed by 40 cycles of 5 sec at 95°C and 30 sec at 58°C/60°C. The results were calculated as the expression of the target gene relative to the expression of the β-actin gene. The sequences of primers used in this study are shown in Table 1.

**Table 1:** Primer sequences.

Protein	Gene	Forward	Reverse
eNOS	*Nos3*	5‘-aaagacaaggcagcagtggaaat	5‘-tccacgatggtgactttggcta
EIF2AK3	*Eif2ak3*	5‘-tgcatatagtggaaaggtgaggt	5‘-cgaggtccgacagctctaac
CHOP	*Ddit3*	5‘-gcgcatgaaggagaaagaac	5‘-tcaccattcggtcaatcaga
PPP1R15A	*Ppp1r15a*	5‘-agctaggactcctctggcaa	5‘-gcttcaggaagggaactgct
ATF6	Atf6	5’-accgtattcttcagggtgc	5’-cactccctgagttcctgctg
GRP-78	*Hspa5*	5‘-acggcagctgctattgctta	5‘-tccatgacacgctggtcaaa
IRE1	*Ire1*	5‘-aaaactacgcctcccctgtg	5‘-gctagatagcgcagggtctc
XBP1u	*Xbp1*	5’-ctgagtccgcagcaggtg	5’-gtccagaatgcccaacagga
XBP1s	*Xbp1* spliced	5’-ctgagtccgcagcaggtg	5’-gtccagaatgcccaacagga
β-actin	*Actb*	5‘-aactgggacgacatggagaaaa	5‘-ggatagcacagcctggatagca

### Western blot analysis

HUVECs were lysed in ice-cold lysis buffer containing 1 mmol/l dithiothreitol, 0.0025% NP40 and a cocktail of proteinase inhibitors. The total protein concentration of the lysate was measured using a kit (BCA Protein Assay, Thermo Fisher). After being heated at 100°C for 5 min, 14 μg of total protein was loaded into each well, separated by 15% SDS-PAGE (Wako), and transferred to nitrocellulose membranes. The membranes were incubated with a mouse monoclonal antibodies against CHOP and ATF6 (Abcam, Cambridge, UK), rabbit polyclonal antibodies against binding immunoglobulin protein (BiP)/GRP78, PERK (Abcam) and phospho-PERK (Thr980) (Bioss, Boston, MA, USA), or rabbit monoclonal antibodies against eNOS and β-Actin (Cell Signaling, Danvers, MA, USA) at 4°C overnight. After being washed, the membranes were incubated with peroxidase-conjugated goat anti-rabbit IgG (Wako) or horseradish peroxidase-linked sheep anti-mouse IgG (GE Healthcare, Buckinghamshire, UK), and then visualized using an ECL system (GE Healthcare).

### Statistical analysis

Data are expressed as the means and SD. Statistical analysis was performed using SAS v.9.3 (SAS Institute, Cary, NC, USA). ANOVA and Student’s *t*-test were used to compare the differences between groups. The results with a *P*<0.05 were considered statistically significant.

## Results

**Fig 1:**
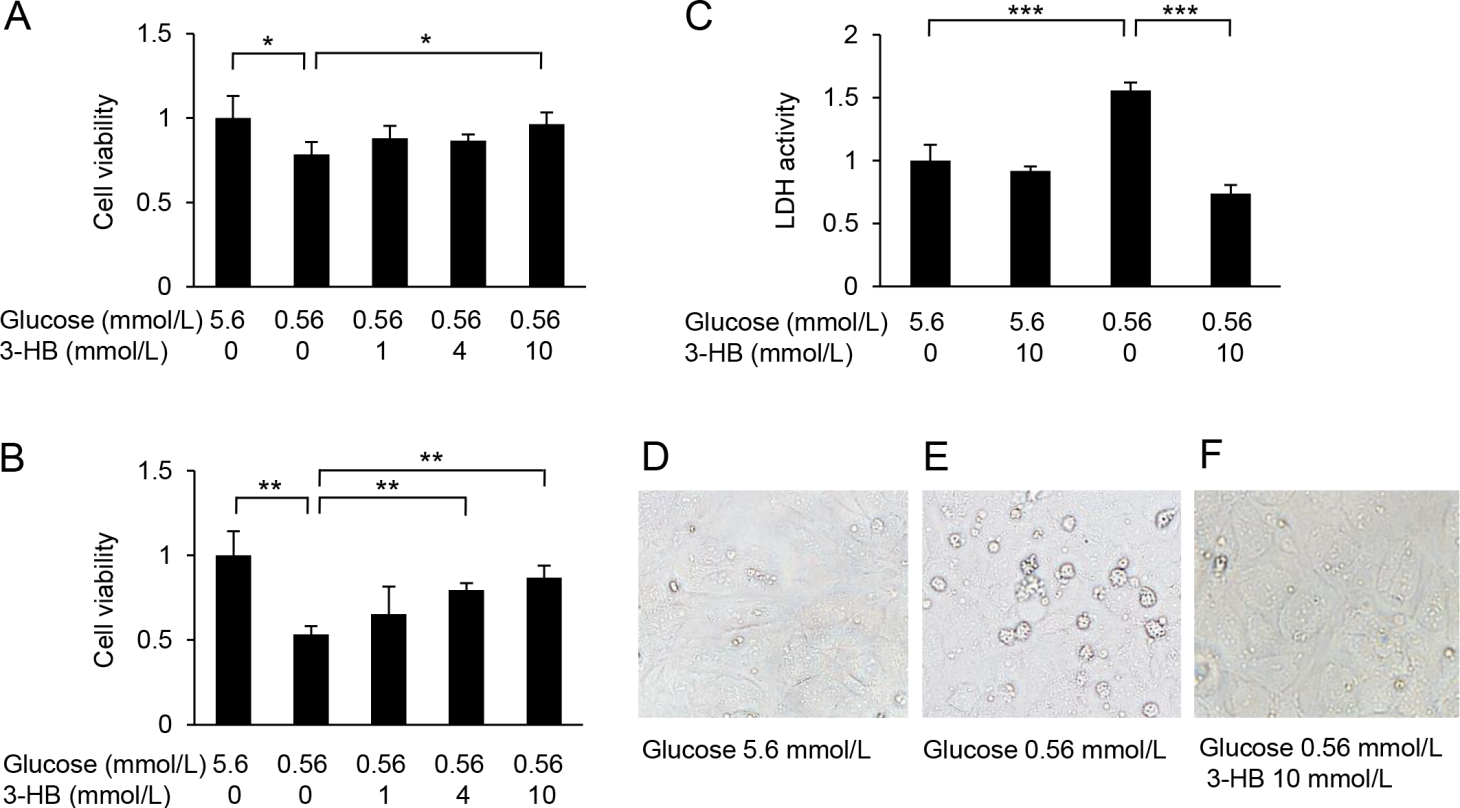
Effects of low-glucose exposure on cell viability and protective effects of 3-hydroxybutyrate (3-HB). Human umbilical vein endothelial cells (HUVECs) were treated with 5.6 or 0.56 mmol/L glucose in the presence of various concentrations of 3-HB (0, 1, 4, 10 mmol/L) for 24 h (A) or 48 h (B). Cell viability was assessed by a CCK-8 assay (A) (B) and an LDH (C) assay. Microscopic images of HUVECs treated for 24 h (D-F). Means and SD, *P<0.05, **P<0.01, ***P<0.001 (n = 4).

**Fig 2:**
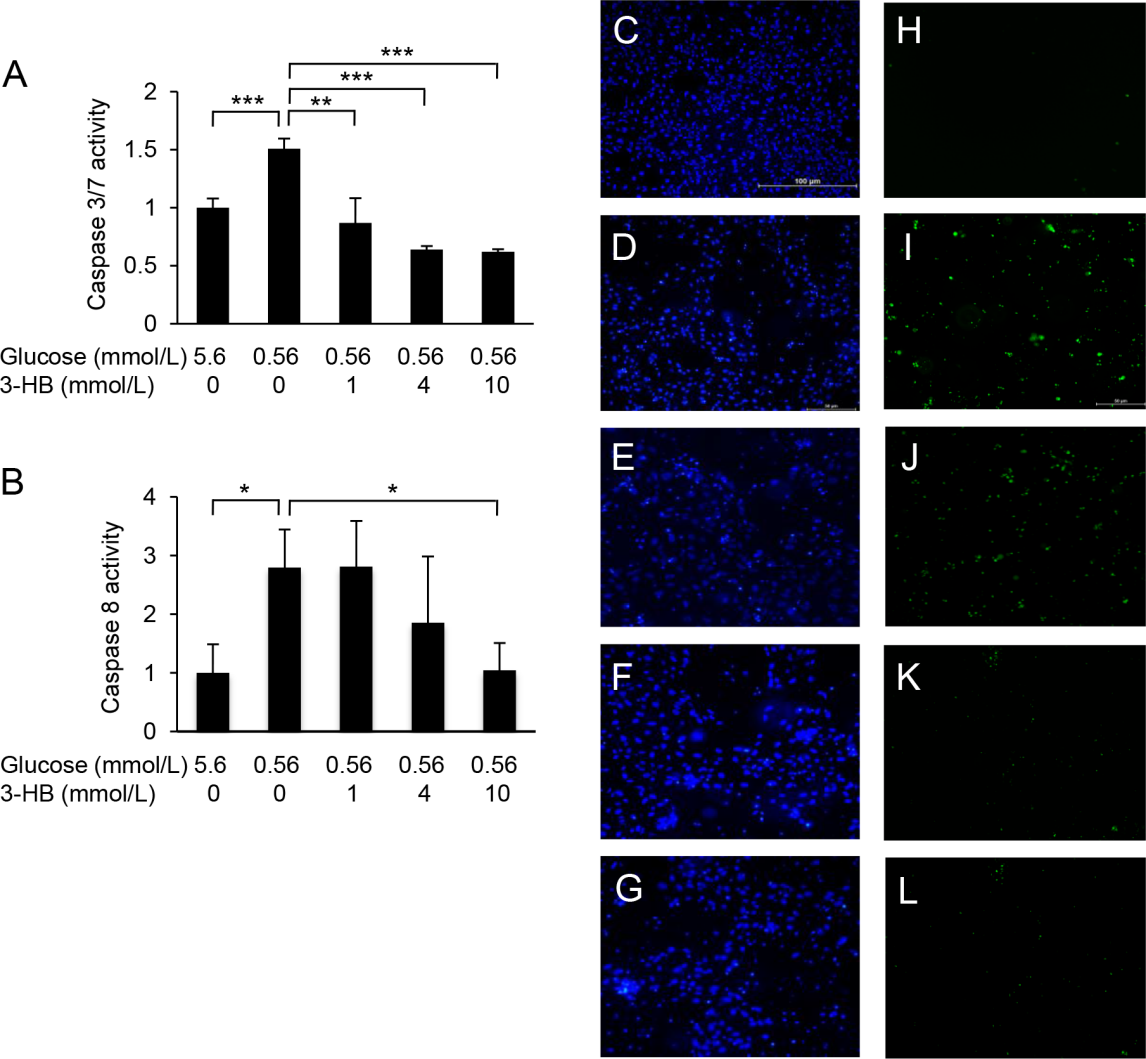
Caspase activation by low glucose and suppressive effect of 3-hydroxybutyrate (3-HB). Caspase-3/7 (A) and caspase-8 (B) activities were measured in HUVECs cultured in low-glucose medium with or without supplementation of 3-HB for 48 h. Results are exppressed as fold increase from control. Means and SD, *P<0.05, **P<0.01, ***P<0.001 (n = 4). Cell nuclei were visualized by Hoechst 33342 staining (blue) (C-G), and caspase-3/7-positive cells were stained green with a fluorogenic substrate (H-L). Bar, 100μm.

**Fig 3:**
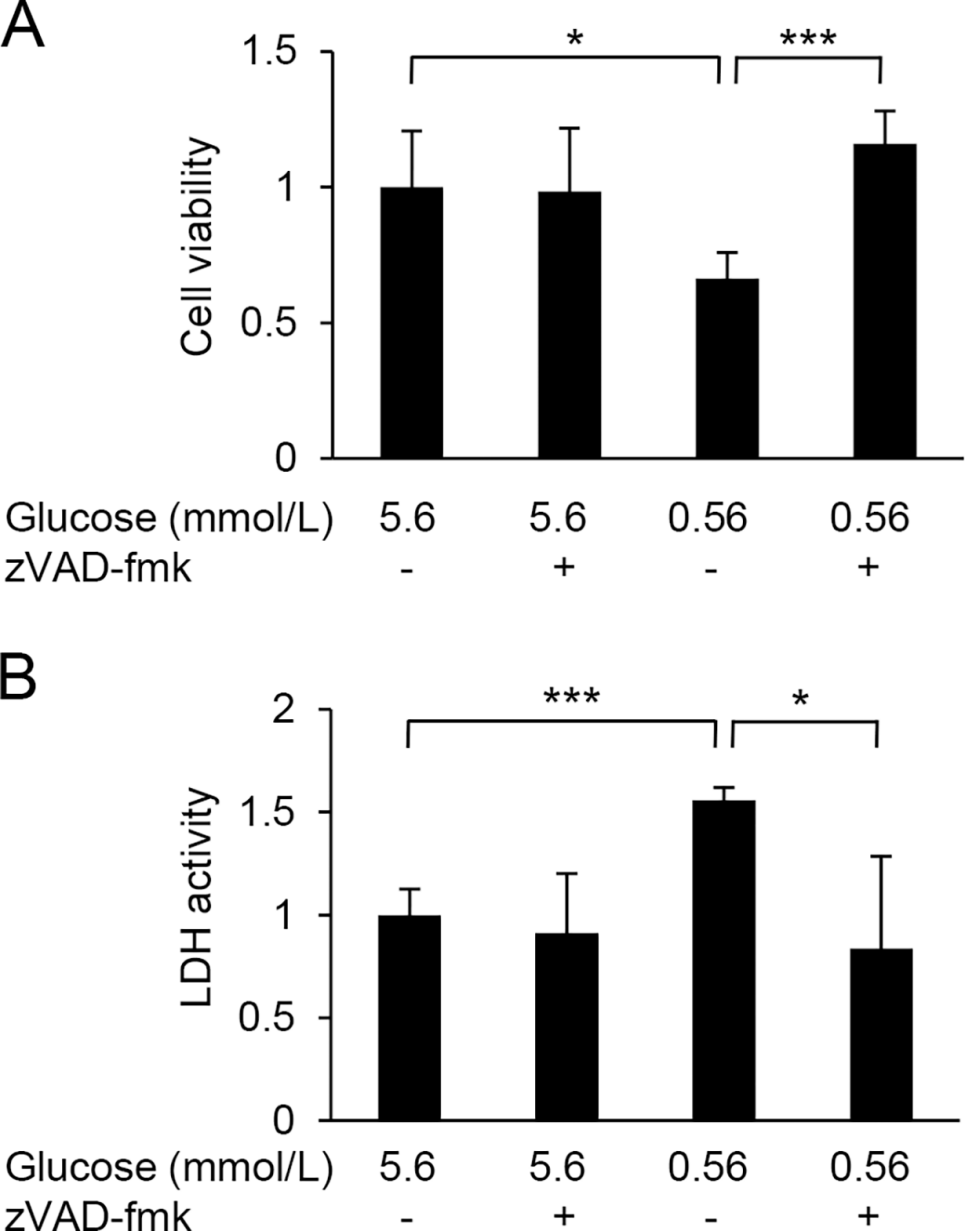
Protective effect of a pan caspase inhibitor on HUVECs exposed to low glucose. HUVECs were treated with 5.6 or 0.56 mmol/L glucose with or without 10 μmol/L zVAD-fmk for 24 h. Cell viability was assessed by the CCK-8 assay (A) and the LDH release assay (B). Means and SD, *P<0.05, **P<0.01, ***P<0.001 (n = 4).

Culture of HUVECs in a low-glucose medium (0.56 mmol/L) for 24 or 48 h resulted in a reduction of cell viability (Fig 1A, 1B and 1E). The low-glucose-induced damage was attenuated by supplementation with 3-HB in a dose-dependent manner (Fig 1A, 1B and 1F). The protective effect of 3-HB was confirmed by the LDH release assay (Fig 1C). Furthermore, 48-h exposure of HUVECs to the low-glucose medium caused the activation of caspase 3/7 and caspase-8, which was suppressed by the addition of 3-HB (Fig 2). Caspase 3/7-positive cells were increased in number after the low-glucose culture, and the increase was inhibited by the supplementation of 3-HB (Fig 2C–2L). The addition of a caspase inhibitor zVAD-fmk to the medium successfully protected HUVECs against the cytotoxic effect of glucose deprivation (Fig 3).

**Fig 4:**
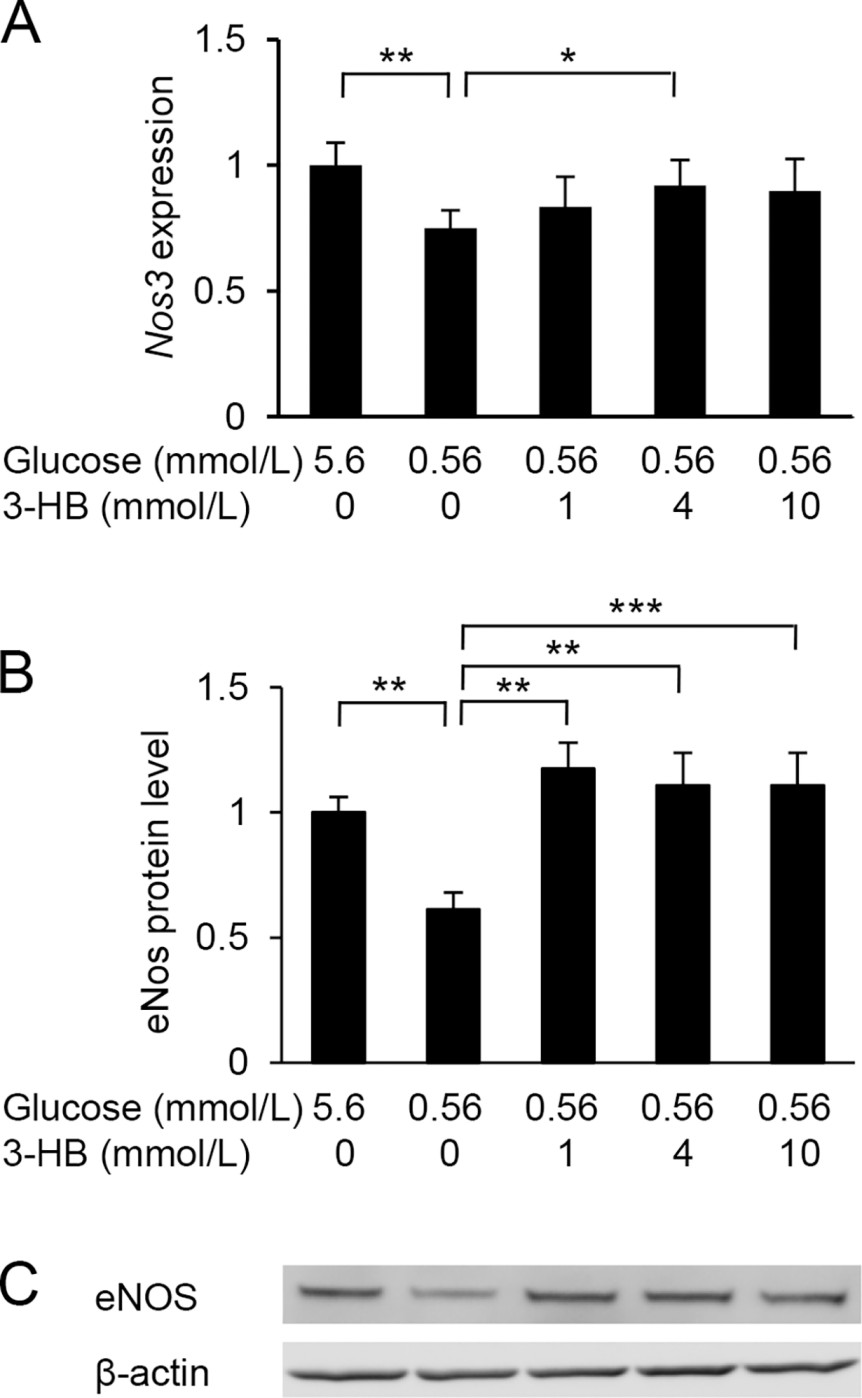
Effect of glucose deprivation on endothelial nitric oxide synthase (eNOS). HUVECs were treated with 5.6 or 0.56 mmol/L glucose in the presence of various concentrations of 3-hydroxybutyrate (3-HB) for 24 h. mRNA (A) and protein levels (B) of eNOS were assessed by real-time PCR and Western blotting, respectively. A representative image of Western blotting is shown (C). Means and SD, *P<0.05, **P<0.01, ***P<0.001.

To assess the effects of low-glucose exposure on vascular endothelial cell function, we measured the gene expression of eNOS (*Nos3*) and eNOS protein levels in HUVECs. After a 24-h incubation in the low-glucose medium, we found reductions in *Nos3* expression and eNOS protein levels, both of which were alleviated by the addition of 3-HB (Fig 4).

**Fig 5:**
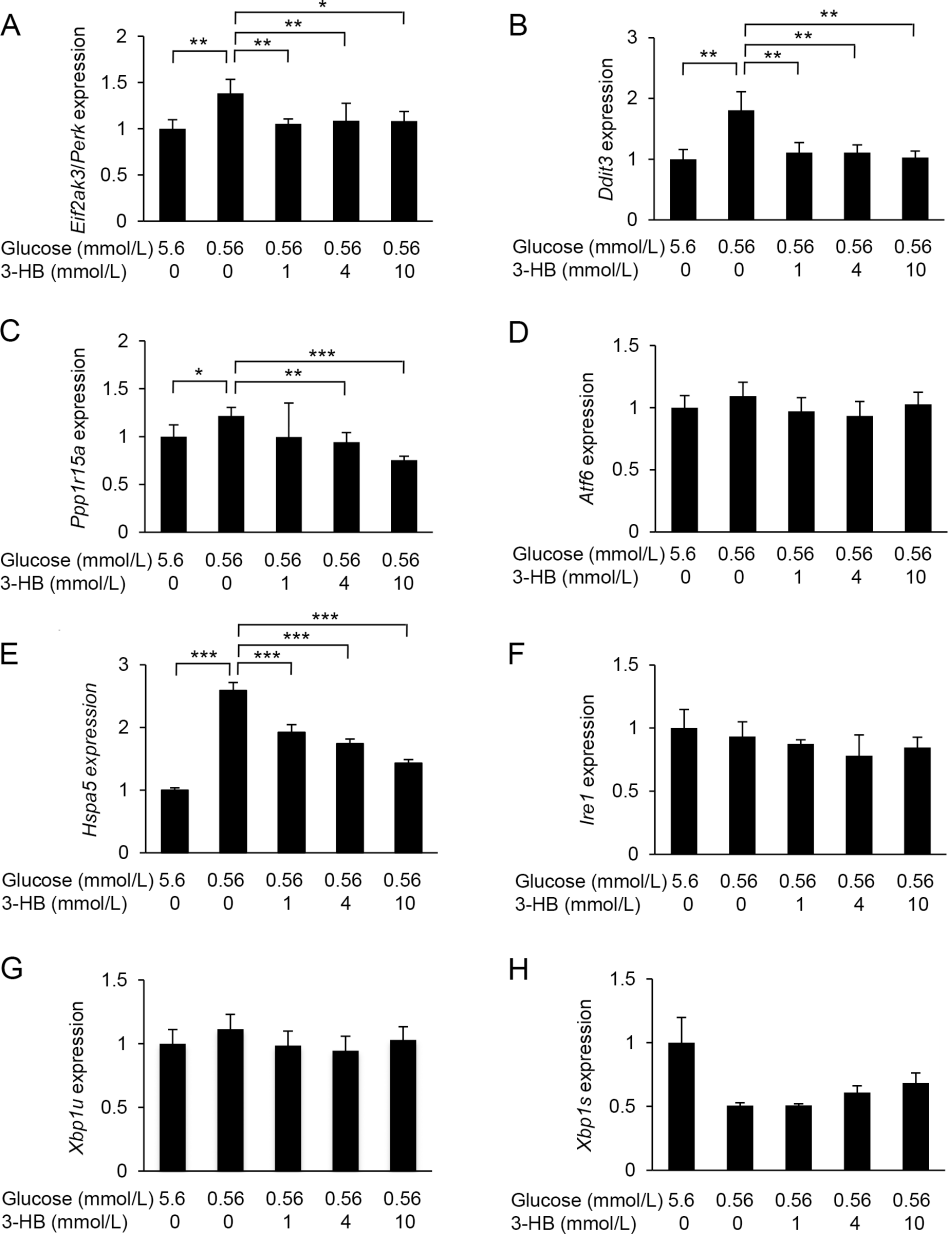
Effects of 3-hydroxybutyrate (3-HB) on the endoplasmic reticulum (ER) stress markers. Expression of ER stress marker genes, *Eif2ak3/Perk* (A), *Ddit3* (B), *Ppp1r15a* (C), *Atf6* (D), *Hsppa5* (E), *Ire1* (F), *XBP1u* (G) and *XBP1s* (H) were assessed in HUVECs treated with 5.6 or 0.56 mmol/L glucose in the presence of various concentrations of 3-HB for 6 h. Means and SD, *P<0.05, **P<0.01, ***P<0.001 (n = 4).

Next, we analysed the gene expression of ER stress-associated genes in low-glucose-treated cells. The expression of *Eif2ak3* encoding PERK, an ER stress sensor, was up-regulated in HUVECs when cultured in the low-glucose medium for 6 h (Fig 5). The expression of two downstream molecules of PERK, i.e., CHOP (*Ddit4*) and GADD34/Ppp1r15a (*Ppp1r15a*), was also increased. The increase in the ER stress markers was dose-dependently suppressed by the addition of 3-HB. The expression of *Atf6* was not significantly changed by the low-glucose culture. However, the expression of the BiP/GRP78 gene (*Hspa5*) was increased by culture in low-glucose medium, and the increase was suppressed by the addition of 3-HB. In contrast, low-glucose exposure did not affect the expression of *Irel*, *and* the splicing of *Xbp1* mRNA was rather reduced by low-glucose culture.

**Fig 6:**
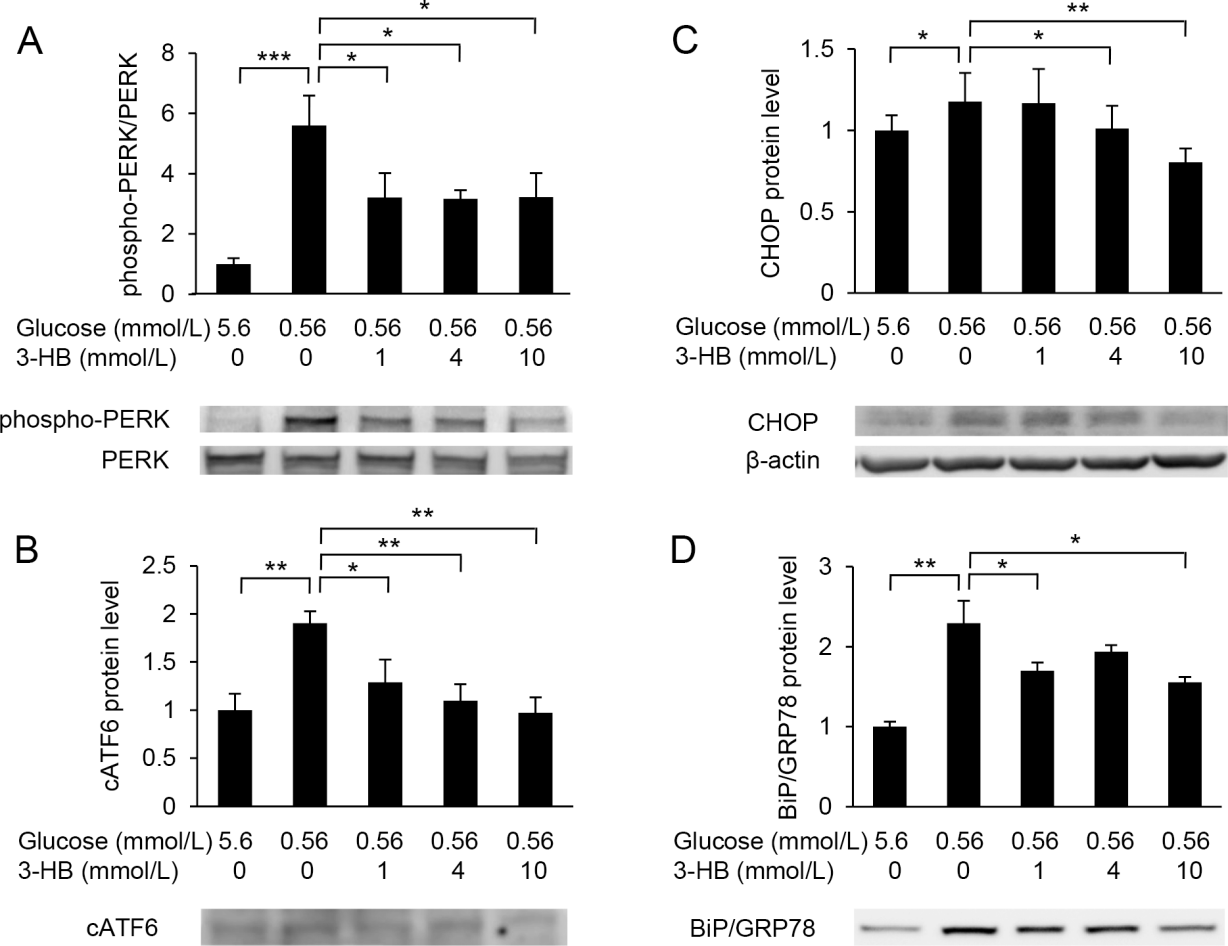
Western blot analysis of ER stress markers. Protein levels of PERK (A), cleaved ATF6 (cATF6) (B), CHOP (C) and Bip/GRP78 (D) were assessed in HUVECs treated with 5.6 or 0.56 mmol/L glucose in the presence of various concentrations of 3-hydroxybutyrate (3-HB) for 24 h. β-actin was used as a loading control. Representative images of Western blotting are shown. Means and SD, *P<0.05, **P<0.01, ***P<0.001 (n = 3).

Western blot analysis showed that low-glucose exposure increased the amount of phospho-PERK and cleaved ATF6 (cATF6) (Fig 6A and 6B). Protein levels of CHOP and BiP/GRP78 were also elevated in HUVECs exposed to low glucose (Fig 6C and 6D). These low-glucose-induced elevations were attenuated by the supplementation of 3-HB (Fig 6).

## Discussion

It has been well described that hyperglycemia-induced endothelial damage is involved in the early stage of atherosclerosis [16, 17]. However, recent studies [1, 18] have shown that hypoglycemia may also play a role in the development of cardiovascular disease, although the mechanism remains to be elucidated. Here, we showed that culture of HUVECs in low-glucose medium resulted in a reduction of viability and impairment of endothelial cell function.

Vascular tone and platelet aggregation are regulated by NO that is generated from L-arginine by endothelial NO synthase (eNOS). Although multiple risk factors are involved in the development and progression of atherosclerosis, a decrease in the amount of bioavailable NO is a common feature of endothelial dysfunction [19]. Thus, the reduction of the eNOS gene (*NOS3*) expression and eNOS protein induced by low-glucose exposure may facilitate the development of atherosclerosis.

It has been suggested that glucose deprivation induces a reduction of protein glycosylation and thereby the accumulation of unfolded proteins [20]. In this study we showed that the low-glucose-induced endothelial cell dysfunction was accompanied by the up-regulation of ER stress marker genes. ER stress activates a signalling network referred to as the unfolded protein response (UPR) to prevent cellular damage. UPR signalling is initiated by three ER-transmembrane proteins that detect ER stress, i.e., PERK, ATF6, and IRE1 [21].

PERK is activated by oligomerization and autophosphorylation when ER stress is increased [22]. In this study PERK gene expression and phosphorylation of PERK protein were up-regulated in HUVECs exposed to low glucose. The expression of downstream factors of the PERK pathway, i.e., CHOP (*Ddit3*/*Gadd153*) and GADD34 (*Ppp1r15a*), was also increased. CHOP is a pro-apoptotic transcriptional factor which mediates ER stress-induced apoptosis [23]. GADD34 exerts feedback inhibition which may be required to enable the cells to produce proteins essential to survive [24].

ATF6 is activated by a sequential action of two proteases S1P and S2P upon ER stress [25]. Although no significant change was observed in the mRNA level of *Atf6*, the amount of cATF6 was increased in HUVECs cultured in low glucose medium. In accordance with this observation, the expression of *Hspa5* which encodes BiP/GRP78, one of the best-characterized ER chaperones induced by ATF6, was significantly increased by the low-glucose culture. A large volume of studies has established that induction of *Hspp5* is a useful marker for ER stress [26].

The third response to increased ER stress is ER-associated degradation (ERAD). This signalling pathway is mediated by IRE1, which is activated by oligomerization and autophosphorylation, similar to PERK. Active IRE1 catalyses the splicing of *Xbp1* mRNA, and spliced XBP1 protein enhances the transcription of ERAD-associated genes [27, 28]. In this study, however, the expression of *Ire1* was not altered, and unexpectedly, *Xbp1* splicing was decreased by glucose deprivation. Thus IRE1 activity seems to be reduced in HUVECs exposed to low glucose. The inadequate capacity of ERAD may be associated with the vulnerability of HUVECs to glucose deprivation.

Moreover, we found activation of caspase-8 and caspase-3/7 by glucose deprivation. Caspase-8 is a key initiator of the caspase cascade leading to apoptosis [29]. Apoptosis mediated by caspase-8 was reported in cancer cells treated with glucose deprivation [30]. On the other hand, caspase-3 is a key effector caspase in the apoptotic signalling cascade [31]. Caspase-7, which shares common substrate specificity with caspase-3, may be also involved in ER stress response induced by glucose deprivation [32]. Furthermore, zVAD-fmk, a cell-permeant pan caspase inhibitor, protected HUVECs against low-glucose-induced cell damage. According to these observations, the reduction in viability of HUVECs exposed to low-glucose medium was likely attributable to ER stress-induced caspase-mediated apoptosis. The observations in this study on low glucose-treated HUVECs are essentially in accordance with previous studies using neurons or cancer cells.

In this study we further assessed the effects of 3-HB supplementation on HUVECs and found that 3-HB dose-dependently attenuated the induction of apoptosis, the reduction of *NOS3*, and the increase of ER stress induced by the low glucose exposure. Ketone bodies can serve as an energy source when glucose availability is limited [13]. 3-HB is readily picked up by the extra-hepatic tissues, independent of insulin actions, and converted to acetyl-CoA, which enters the mitochondria and is oxidized in the Krebs cycle to generate ATP. Thus, the induction of ER stress by glucose deprivation may be attributable not only to the insufficient glycosylation but to the energy deficiency. The increase in energy supply by 3-HB may be associated with the attenuation of low-glucose-induced ER stress, although the precise mechanism is still unknown.

Ketone bodies are important sources of energy for the brain, which is incapable of using FFA as fuel [33]. A high fat, low carbohydrate ketogenic diet is an established nonpharmacological treatment for epilepsy in childhood [34,35]. Several studies have shown the beneficial effects of a ketogenic diet or ketone bodies on ischemic stroke and Alzheimer’s disease [36–38]. In rats, systemic administration of 3-HB was shown to prevent hypoglycemia-induced neuronal death [39]. Although the precise mechanism is not known, 3-HB may attenuate low-glucose-induced ER stress through the increase in energy supply.

The possible protective effects of ketone bodies on cardiovascular disease presently attract a great deal of interest because the EMPA-REG OUTCOME study recently demonstrated a remarkable effect of the SGLT2 inhibitor empagliflozin on cardiovascular and renal outcomes [13, 40]. SGLT2 inhibitors increase urine glucose excretion and thereby result in the improvement of plasma glucose levels and body weight accompanied by an increase in plasma ketone bodies [41]. Although excessive production of ketone bodies may lead to dangerous ketoacidosis, mild to moderate elevation of ketone bodies in plasma could result in an improvement in energy metabolism in diabetic patients with insulin resistance. The exact mechanism of the beneficial effect of empagliflozin is still unclear and may be multifaceted. However, the elevation of plasma ketone bodies has been implicated as a mechanism by which empagliflozin prevented cardiorenal events [41], although the concentration of ketone bodies in treated patients was much lower than the doses used in this study.

In conclusion, culture of HUVECs in low-glucose medium resulted in an increase in ER stress leading to the induction of endothelial dysfunction and caspase-mediated apoptosis. These cellular responses caused by glucose deficiency were efficiently suppressed by supplementation with 3-HB, a major component of plasma ketone bodies. Thus, hypoketonemic hypoglycemia induced by insulin injection or insulin secretagogue administration may be associated with a higher risk of cardiovascular events when compared with hyperketonemic hypoglycemia induced by starvation or SGLT2 inhibitor therapy. This may also be the case for other organs vulnerable to hypoglycemia, such as the brain.
